# Chronic and acute thermal stressors have non-additive effects on fertility

**DOI:** 10.1098/rspb.2024.1086

**Published:** 2024-09-18

**Authors:** Natalie Pilakouta, Daniel Allan, Ellie Moore, Alison A. Russell

**Affiliations:** ^1^ Centre for Biological Diversity, School of Biology, University of St Andrews, St Andrews KY16 9TH, UK

**Keywords:** burying beetle, climate change, hatching success, fecundity, parental care, reproduction

## Abstract

Climate change is driving both higher mean temperatures and a greater likelihood of heatwaves, which are becoming longer and more intense. Previous work has looked at these two types of thermal stressors in isolation, focusing on the effects of either a small, long-term increase in temperature or a large, short-term increase in temperature. Yet, a fundamental gap in our understanding is the combined effect of chronic and acute thermal stressors and, in particular, its impact on vital processes such as reproduction. Here, we investigated the independent and interactive effects of higher constant temperatures and short-term heatwave events on reproductive success and offspring fitness in an insect study system, the burying beetle *Nicrophorus vespilloides*. We found a substantial reduction in key fitness traits (fecundity, hatching success and offspring size) after exposure to both a heatwave and higher constant temperatures, but not after exposure to only one of these thermal stressors. This indicates that the effects of chronic and acute thermal stressors are amplified when they act in combination, as is very likely to occur in natural populations. Our findings, therefore, suggest that, by not considering the potential multiplicative effects of different types of thermal stressors, we may be underestimating the effects of climate change on animal fertility.

## 1. Introduction

Global climate change is a pressing issue with profound implications for biodiversity. It is driving both higher mean temperatures and a higher likelihood of heatwaves, which are becoming more intense, more variable and longer in duration [1,2]. Experimental studies have typically looked at these two components of climate change in isolation, focusing on the effects of either a small, long-term increase in temperature or a large, short-term increase in temperature, representing a simulated heatwave (e.g. [3–9]). Short-term heatwaves can impose severe thermal stress with dramatic consequences for survival and reproduction [7,8]. Long-term higher constant temperatures can also negatively affect fitness by altering the baseline thermal conditions experienced by organisms, affecting their physiological performance [10,11]. Understanding how organisms respond to concurrent exposure to both chronic (long-term) and acute (short-term) thermal stressors is critical for predicting their vulnerability to climate change.

**Figure 1.**
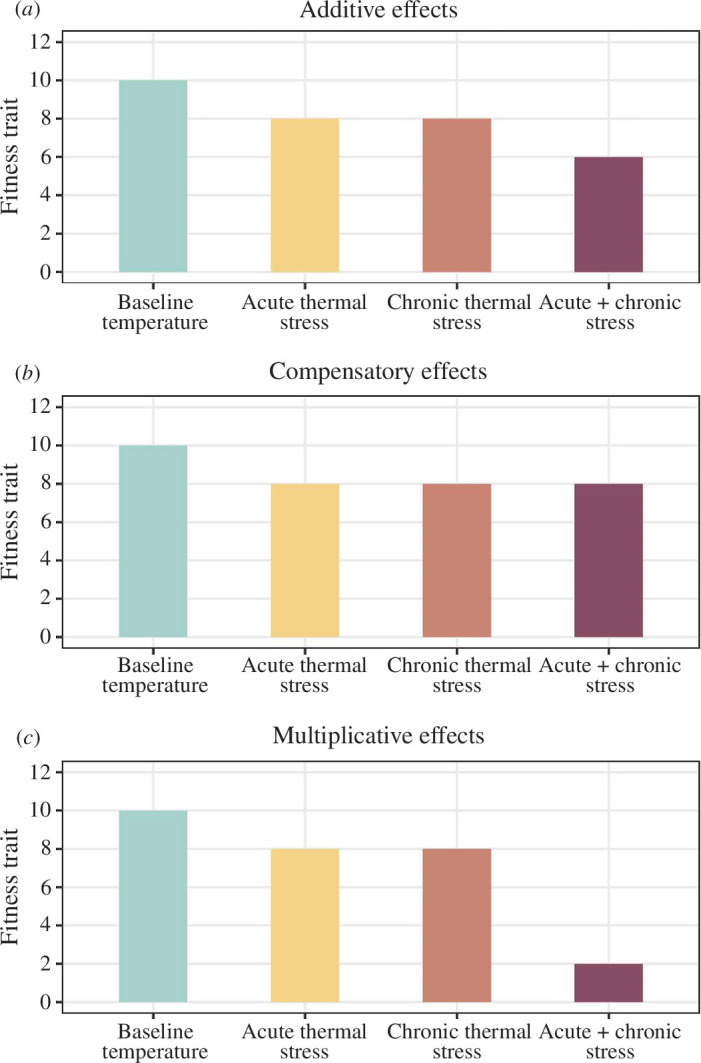
Conceptual diagram illustrating different scenarios of the independent and combined effects of long-term higher constant temperatures (chronic thermal stressor) and short-term heatwaves (acute thermal stressor). If the effects of these different types of stressor are additive (*a*), we expect that the sum of the independent effects of these thermal stressors would correspond to the combined effect. On the other hand, if these effects are compensatory (*b*), the combined effect of these stressors would be less than the sum of the independent effects. Lastly, if these effects are multiplicative (*c*), the combined effect of these stressors would be greater than the sum of the independent effects.

Despite extensive research on the individual effects of chronic and acute thermal stressors on fitness, there is a fundamental gap in our understanding of how these stressors interact when experienced simultaneously. One possibility is that the effects of long-term higher constant temperatures and short-term heatwaves on fitness traits are additive. Under this scenario, we would expect the sum of the independent effects of these thermal stressors to be similar to the effect of simultaneous exposure to these stressors (figure 1). Alternatively, these effects may be non-additive if they act antagonistically or synergistically (i.e. compensatory or multiplicative effects, respectively). If these effects are compensatory, their combined effect will be less than expected based on their individual effects [12]. If these effects are multiplicative, their combined effect will be more than expected based on their individual effects [13]. Evidence for this last scenario would suggest that current literature may be underestimating the effects of climate change on natural populations by only studying the independent effects of sustained elevated temperatures and heatwave events. Answering this question is thus crucial for accurately predicting the ecological consequences of climate change, including its impact on vital processes, such as reproduction.

Animal reproduction is highly sensitive to thermal stress: elevated temperatures can affect a range of reproductive traits, from gonad development to gamete quantity and quality, to fertilization success and offspring performance (e.g. [3–6,14–17]). Even a slight reduction in fertility can have dramatic consequences for population survival, so there is a pressing need to better understand how climate change will affect reproduction in natural populations [18].

Here, we address this knowledge gap by investigating the combined effects of higher constant temperatures and short-term heatwaves on reproductive success and offspring fitness in an insect study system, the burying beetle *Nicrophorus vespilloides*. This species breeds on carcasses of small vertebrates and has facultative biparental care that includes food provisioning, carcass maintenance through the deposition of antimicrobial substances and brood defence against predators and conspecifics [19]. Females typically spend more time provisioning food for the larvae and stay on the carcass for longer than males [20–24]. Previous work has shown that simulated heatwaves occurring during breeding have detrimental effects for parental care, reproductive success and offspring fitness in this species [8].

**Figure 2.**
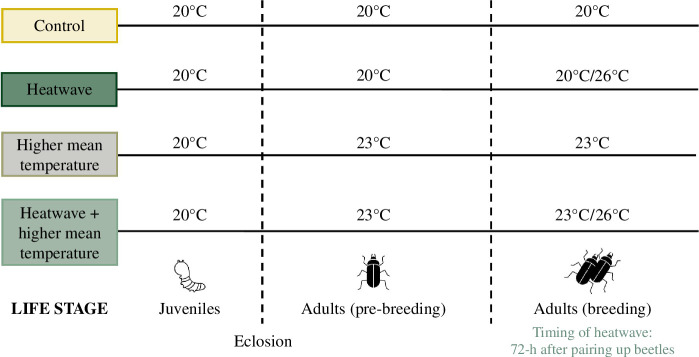
Visualization of the experimental design used to assess the independent and combined effects of long-term higher constant temperatures (chronic thermal stressor) and short-term heatwave events (acute thermal stressor) on reproduction. The timing of the 26°C heatwave was 72 h post-mating and its duration was 72 h (*sensu* [8]).

Our experimental design included (i) a control treatment where beetles were kept at a constant temperature (20°C) throughout the experiment, (ii) beetles kept at 20°C but exposed to a 3 day heatwave (26°C) during breeding, (iii) beetles kept at a higher constant temperature (23°C) post-eclosion and during breeding, and (iv) beetles kept at a higher constant temperature (23°C) post-eclosion and exposed to a 3 day heatwave (26°C) during breeding (figure 2). By examining the independent and combined effects of chronic and acute thermal stressors on parental care, reproductive success and offspring fitness, our study can, therefore, reveal whether these effects are additive, compensatory or multiplicative.

## 2. Methods

### (a) Study species

Burying beetles (*N. vespilloides*) breed on vertebrate carcasses and provide elaborate parental care. When a carcass is found, parents bury it in the soil, remove its fur or feathers and deposit antimicrobial secretions on it to prevent bacterial and fungal growth [19]. They lay eggs in the soil around the carcass approximately 2 days after mating [25]. The eggs hatch a few days later and the larvae crawl to the carcass. Larvae can self-feed in a crater created by the parents on the carcass, but the parents also provision larvae with predigested carrion [26]. Larvae disperse from the carcass 5–7 days after hatching, which corresponds to the end of the parental care period and to offspring independence.

### (b) Animal husbandry

We used second- and third-generation beetles from an outbred laboratory population maintained at the University of St Andrews. These lines originated from wild-caught beetles collected in Fife, Scotland in August and September 2023. Beetles were housed individually in transparent plastic containers (12 × 8 × 2 cm) filled with moist soil and kept at 20°C in cooled incubators (LMS Series 1A Model 201 NP). They were fed small cubes of raw organic beef twice a week.

### (c) Experimental design and procedures

Our experimental design included (i) a control treatment where beetles were kept at a constant temperature (20°C) throughout the experiment, (ii) beetles kept at 20°C but exposed to a 3 day heatwave (26°C) 72 h after being paired with a partner, (iii) beetles kept at a higher constant temperature (23°C) post-eclosion and during breeding, and (iv) beetles kept at a higher constant temperature (23°C) post-eclosion and exposed to a 3 day heatwave (26°C) 72 h after being paired with a partner (figure 2). To ensure that our simulated heatwave was ecologically relevant, we used 26°C for 3 days, which is representative of the typical duration and upper temperature limit of heatwaves experienced in central Scotland in recent years (*sensu* [8]). Data for the four treatment groups were collected concurrently using different incubators. Beetles were kept in two cooled incubators set to a constant temperature of 20 or 23°C and a 16 h:8 h light cycle. For treatment groups that were exposed to a heatwave, we used a third incubator set to a constant temperature of 26°C and the same light cycle. We also used a saturated solution of magnesium chloride hexahydrate salt to maintain the humidity inside the incubator at 50% (±10%). Humidity levels were monitored using Fisherbrand™ Traceable™ Jumbo Thermo-Humidity Meters.

We mated unrelated virgin males and females at least 10 days after eclosion, which is when they reach sexual maturity. Each experimental pair was placed in a transparent plastic container (17 × 12 × 6 cm) filled with 1 cm of moist soil and a freshly thawed mouse carcass of a standardized size (22‒25 g). We set up a total of 200 matings across the four treatments. Our sample sizes for pairs that had at least one larva surviving to independence were *n* = 33 for the control treatment (constant temperature of 20°C), *n* = 31 for the heatwave treatment (26°C for three days), *n* = 25 for the treatment with a higher constant temperature (23°C) and *n* = 25 for the treatment with a higher constant temperature (23°C) and heatwave (26°C for 3 days).

We counted eggs by checking the underside of each breeding box twice a day until larvae started hatching. This is the least disruptive method for estimating clutch size in this species [27], and previous work found a strong correlation between the number of eggs counted from the underside of the breeding box and the actual number of eggs laid [28]. Shortly before the dispersal stage, we counted the number of unhatched eggs, which was subtracted from the total number of eggs laid, and the difference was then divided by the total number of eggs to determine hatching success.

To estimate the duration of care by each parent, we checked the containers daily in the morning and in the afternoon to determine whether parents were present on the carcass or away from the brood in the soil. Parents that were away for more than two consecutive checks were deemed to have abandoned the brood and were removed from the boxes to prevent infanticide [24]. At the dispersal stage, when offspring become independent, we recorded the date and the number of surviving larvae. The number of days between beetles being paired up and the larvae dispersing was the breeding bout length. We also measured total brood mass at dispersal, using an Ohaus PR124 analytical balance with a precision of 0.0001 g. We divided the total brood mass by the number of larvae in the brood to calculate the average offspring mass.

### (d) Data analysis

All analyses were performed using R version 4.3.1 [29]. We used packages ‘ggplot2’, ‘cowplot’ and ‘wesanderson’ for generating figures [30]. Treatment was used as an explanatory variable in each of the models described below. We used linear models to examine effects on total brood mass and average offspring mass, which were continuous traits with normally distributed random errors. For discrete traits, we used generalized linear models fitted with a negative binomial error distribution (number of eggs laid) or a quasi-Poisson error distribution (breeding bout length and parental care duration) to account for overdispersion. For proportion data (hatching success), we used generalized linear models fitted with a quasi-binomial distribution. For variables where treatment had a statistically significant effect, we also conducted *post hoc* pairwise comparisons using the ‘emmeans’ package [31] to determine which treatments differed from each other. The Tukey method within the ‘emmeans’ package was used to adjust *p*-values due to multiple comparisons.

If our results show that experiencing only acute thermal stress or only chronic thermal stress has no significant effect on a given fitness trait, whereas there is a significant effect when these stressors act in combination, this would be evidence for multiplicative effects (figure 1). On the other hand, if we find that the independent and combined thermal stressors all have a significant effect on fitness, this would suggest compensatory or additive effects (figure 1). Distinguishing between these two scenarios would require comparing effect sizes across the three thermal treatments to determine whether these are the same (compensatory effects) or whether the effect size for the combined thermal stressors is similar to the sum of the effect sizes of the individual thermal stressors (additive effects). There are, of course, various other possible scenarios where, for example, none of the independent or combined thermal stressors has any effect on the fitness traits being studied.

## 3. Results

We found that short-term simulated heatwaves, but not higher constant temperatures, negatively affected some aspects of reproduction (i.e. brood mass and male parental care; table 1). Interestingly, we also found evidence that the combined effects of higher constant temperatures and heatwaves were non-additive for a number of key fitness traits (fecundity, hatching success and offspring size). More specifically, this combined effect led to a greater fitness reduction than would be expected based on the effects of the two independent treatments (table 1), suggesting that the effects of chronic and acute thermal stressors are multiplicative.

**Table 1.** Results of *post hoc* pairwise comparisons using the ‘emmeans’ package. We compared the control treatment (constant temperature of 20°C) with the three thermal stress treatments (heatwave only, higher constant temperature only or combination of heatwave and higher constant temperature). The Tukey method was used to adjust *p*-values due to multiple comparisons. Statistically significant *p*-values (<0.05) are indicated in bold.

	control – heatwave	control – higher constant temperature	control – heatwave + higher constant temperature
*reproductive success*			
fecundity	estimate = 0.01 ± 0.16, *z* = 0.04 *p* > 0.99	estimate = 0.26 ± 0.19, z = 1.36 *p* = 0.52	estimate = 0.62 ± 0.19, *z* = 3.33 ***p* = 0.005**
brood mass	estimate = 0.96 ± 0.33, *t* = 2.91 ***p* = 0.02**	estimate = −0.07 ± 0.35, *t* = −0.20 *p* > 0.99	estimate = 1.12 ± 0.35, *t* = 3.17 ***p* = 0.01**
length of breeding bout	estimate = 0.07 ± 0.03, *z* = 2.02 *p* = 0.18	estimate = 0.14 ± 0.04, *z* = 4.14 ***p* < 0.001**	estimate = 0.14 ± 0.04, *z* = 4.14 ***p* < 0.001**
*parental care*			
male duration of care	estimate = 0.13 ± 0.04, *z* = 3.70 ***p* = 0.001**	estimate = 0.07 ± 0.04, *z* = 1.72 *p* = 0.31	estimate = 0.04 ± 0.04, *z* = 0.98 *p* = 0.76
female duration of care	estimate = 0.11 ± 0.04, *z* = 2.57 *p* = 0.050	estimate = 0.05 ± 0.05, *z* = 1.01 *p* = 0.75	estimate = 0.06 ± 0.05, *z* = 1.10 *p* = 0.69
*offspring fitness*			
hatching success	estimate = −0.27 ±0.54, z = −0.49 *p* = 0.96	estimate = 0.44 ± 0.55, *z* = 0.80 *p* = 0.86	estimate = 1.33 ± 0.51, *z* = 2.60 ***p* = 0.046**
offspring size	estimate = 0.01 ± 0.01, *t* = 1.48 *p* = 0.46	estimate = −0.02 ± 0.01, *t* = −1.56 *p* = 0.41	estimate = 0.03 ± 0.01, *t* = 3.27 ***p* = 0.008**

### (a) Effects on reproductive success

**Figure 3.**
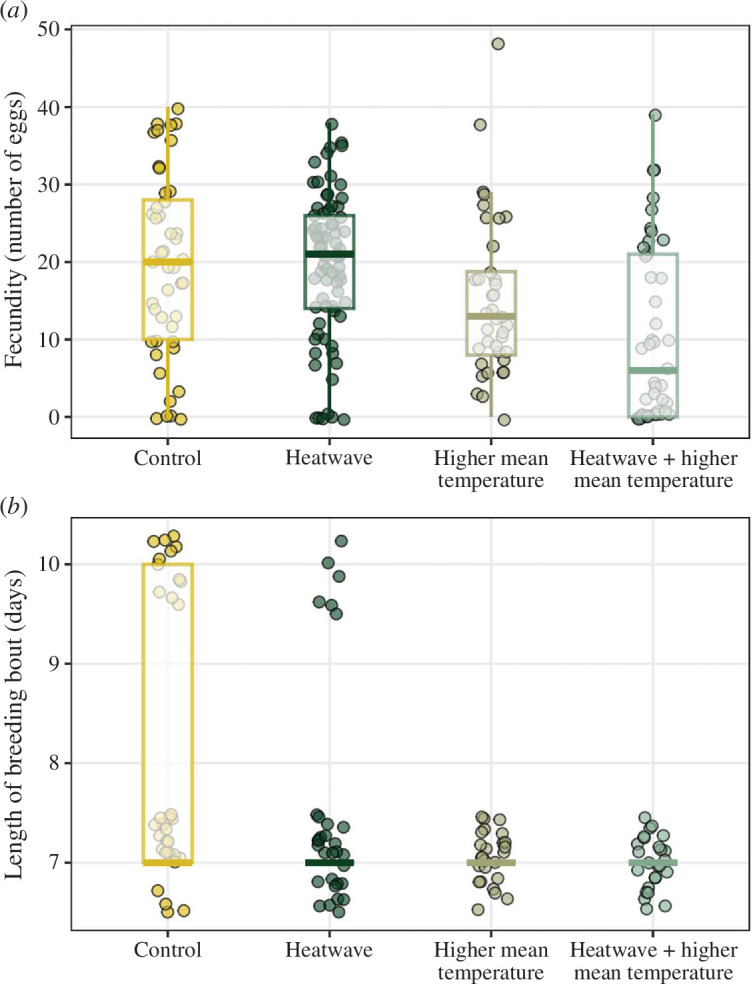
Boxplots of fecundity (*a*) and breeding bout length (*b*) across the four treatment groups. The lower and upper hinges of the box correspond to the first and third quartiles, respectively. The lower and upper whiskers extend from the hinge to the smallest and largest value no further than 1.5 × interquartile range from the hinge, respectively.

There was no evidence for an effect on fecundity (number of eggs laid) for parents that were exposed only to a heatwave or only to a higher constant temperature (table 1). However, parents that were exposed to both higher constant temperatures and a simulated heatwave suffered a substantial reduction in fecundity compared with the control treatment (LR *χ^2^* = 14.9, *p* = 0.002; table 1 and figure 3*a*). We also found that parents exposed to a heatwave or to both a higher constant temperature and a heatwave had a lower total brood mass than the control treatment (LR *χ^2^* = 32.5, *p* < 0.001; table 1). Lastly, parents exposed to a higher constant temperature, with or without a heatwave, had shorter breeding bouts (LR *χ^2^* = 24.9, *p* < 0.0001; table 1 and figure 3*b*).

### (b) Effects on parental care

We found that thermal stress led to a reduction in the duration of parental care provided by males (LR *χ^2^* = 14.8, *p* = 0.002) but not by females (LR *χ^2^* = 6.81, *p* = 0.078). More specifically, males exposed to a heatwave tended to abandon their broods earlier (table 1). Similarly, there was a marginally nonsignificant trend of earlier abandonment by females exposed to a heatwave (*p* = 0.050; table 1).

### (c) Effects on offspring fitness

**Figure 4.**
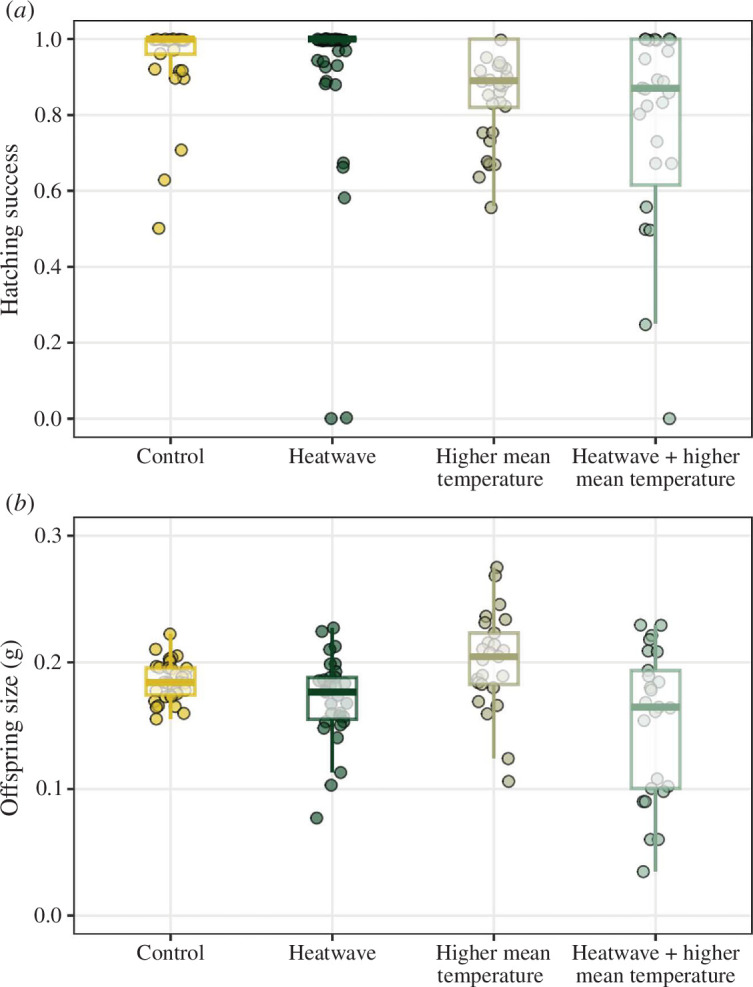
Boxplots of hatching success (*a*) and offspring size at independence (*b*) across the four treatment groups. The lower and upper hinges of the box correspond to the first and third quartiles, respectively. The lower and upper whiskers extend from the hinge to the smallest and largest value no further than 1.5 × interquartile range from the hinge, respectively.

There was no effect on the hatching success or size of offspring whose parents were exposed only to a heatwave or only to a higher constant temperature (table 1). However, offspring whose parents were exposed to both higher constant temperatures and a simulated heatwave suffered reduced hatching success (LR *χ^2^* = 13.2, *p* = 0.004; figure 4*a*) and they were smaller at the stage of offspring independence (sum of squares, SS = 0.03, *F* = 7.57, *p* = 0.0001; figure 4*b*).

## 4. Discussion

Predicting the effects of multiple stressors is currently one of the most significant challenges in the field of ecology [32–34]. Understanding the cumulative effects of multiple stressors on organisms is essential for managing their ecological impacts [12,34,35]. Previous work has examined the non-additive effects of stressors such as temperature, salinity, turbidity, hypoxia, toxin exposure, low food availability and disease [12,13,36,37]. However, to our knowledge, this is the first study to test the independent and interactive effects of different types of thermal stressors that organisms are likely to experience under climate change. We show evidence for multiplicative effects of higher constant temperatures and short-term heatwaves on reproductive success and offspring fitness. More specifically, we found a substantial reduction in a number of key fitness traits (fecundity, hatching success and offspring size) when parents were exposed to both higher constant temperatures and a heatwave, but not when they were exposed to only one of these thermal stressors. Our findings highlight the importance of testing the combined effects of different thermal stressors and suggest that we may currently be underestimating the effects of climate change.

Two of the fitness traits where we observed multiplicative effects of chronic and acute thermal stressors were fecundity and hatching success, which have previously been shown to be highly sensitive to increased temperature across diverse taxa (e.g. [38–40]). The observed reduction in fecundity and hatching success in our study could be due to the effects of thermal stress on males, females or both sexes. Previous work in other study systems has shown that temperature can have sex-specific effects depending on the species but also depending on the type of thermal stress (e.g. [41–46]). In this study, increased temperature may have influenced male and/or female mating behaviour, and the resulting mating success (47). Thermal stress may have also been detrimental for gamete-related traits in males (e.g. sperm production and sperm quality), females (e.g. egg production and egg quality) or both. Thermal effects on gamete quality could have, in turn, negatively affected fertilization success by influencing sperm viability and motility, sperm–egg recognition and binding, sperm penetration of the egg, egg activation, and other sperm–egg interactions [48]. The behavioural and physiological mechanisms underlying differences in fecundity and hatching success across thermal treatments were beyond the scope of the present study, but this would be a fruitful avenue for future research.

There was also evidence of multiplicative effects on offspring mass at the dispersal stage, which corresponds to the end of the parental care period in *N. vespilloides*. Offspring mass at independence is a crucially important fitness component in this species. Larvae do not feed after dispersal and before eclosion, so larval mass determines adult size [49]. In turn, adult size influences lifespan, fecundity and the likelihood of acquiring a carcass for breeding [50–53]. The smaller size of offspring at independence could have been a result of a reduction in parental care, which includes provisioning food to the larvae, thus improving their growth rate [54]. Physiological stress and higher metabolic costs of care due to increased temperature may lead to a reduced capacity to provide parental care, especially in ectotherms (e.g. [55]). Although there was no evidence for a reduction in the duration of male or female care when parents were exposed to both higher constant temperatures and a short-term heatwave, we do not have any information on the quality of care provided during that time. Given that we did not conduct behavioural observations, we cannot exclude the possibility that parents varied in the amount of care they provided, even if there were no differences in how long they stayed with the brood (e.g. [24]). An alternative explanation is that an accelerated carcass decomposition rate at higher temperatures reduced the amount of resources available to the offspring [56]. Heat stress experienced by the offspring may have also directly impaired their growth rate by increasing metabolic costs [57]. These explanations are not mutually exclusive, and it is possible that any direct effects of thermal stress on offspring fitness were exacerbated by a faster rate of carcass decomposition or a reduction in the amount of care provided. Regardless of the underlying cause, our results indicate that concurrent exposure to both chronic and acute thermal stressors has major consequences for offspring fitness.

Another interesting finding was that simulated heatwaves, but not higher constant temperatures, negatively affected some aspects of reproduction: total brood mass, male duration of care and female duration of care. This suggests that short periods of exposure to more extreme temperatures may be more stressful to organisms than long-term exposure to a smaller increase in temperature. Indeed, the only reproductive trait that was affected by higher constant temperatures was breeding bout length, which refers to the amount of time between the parents mating and the offspring dispersing from the carcass. Unsurprisingly, we found that breeding bouts were shorter at higher constant temperatures (with or without a heatwave). This is in line with previous studies showing faster developmental rates in warmer environments, especially in ectotherms (e.g. [58–61]).

In this study, we used a maximum temperature (26°C) that is under the thermal fertility limit for this species, because we wanted to have a high enough breeding success to measure a range of reproductive traits. Nevertheless, under climate change, the combination of higher mean temperatures and short-term heatwave events in natural populations is likely to push organisms past their thermal fertility limit, which could lead to complete breeding failure (e.g. [17]). We indeed have preliminary data showing that in a warming scenario with a new baseline temperature of 23°C, a heatwave-induced temperature increase of 6°C reduces the likelihood of successful breeding to zero in this species. Out of 30 matings we set up, 33% failed to lay any eggs, 73% had no hatching success and 100% had zero larvae surviving to the dispersal stage, following exposure to a 29°C heatwave (electronic supplementary material, figure S1).

## 5. Conclusion

Our study provides novel insights into the effects of climate change on reproduction, by showing that the effects of chronic and acute thermal stressors are amplified when they act in combination. We expect such non-additive effects to be common in natural populations where organisms are bound to be exposed to different types of thermal stressors at the same time [13]. Given that our current knowledge is based on studies examining the effects of sustained elevated temperatures and short-term heatwaves separately, our findings suggest that we may be underestimating the effects of climate change on fertility and population viability. We therefore strongly encourage future studies that investigate whether these effects are additive, compensatory or multiplicative in other taxa. In sum, this work advances our understanding of the complex interplay between chronic and acute thermal stressors in shaping organismal responses. This information is crucial for improving our ability to make informed predictions about the ecological consequences of climate change.

## Data Availability

All relevant data, code and the associated README file can be found attached in the online electronic supplementary material [62].
